# The discovery and characterization of AeHGO in the branching route from shikonin biosynthesis to shikonofuran in *Arnebia euchroma*

**DOI:** 10.3389/fpls.2023.1160571

**Published:** 2023-04-25

**Authors:** Ruishan Wang, Changzheng Liu, Chaogeng Lyu, Jiahui Sun, Chuanzhi Kang, Ying Ma, Xiufu Wan, Juan Guo, Linyuan Shi, Jinye Wang, Luqi Huang, Sheng Wang, Lanping Guo

**Affiliations:** State Key Laboratory Breeding Base of Dao-di Herbs, National Resource Center for Chinese Materia Medica, China Academy of Chinese Medical Sciences, Beijing, China

**Keywords:** shikonin derivatives, shikonofuran derivatives, *Arnebia euchroma*, biosynthesis, cinnamyl alcohol dehydrogenase, AeHGO, metabolic regulation

## Abstract

Shikonin derivatives are natural naphthoquinone compounds and the main bioactive components produced by several boraginaceous plants, such as *Lithospermum erythrorhizon* and *Arnebia euchroma*. Phytochemical studies utilizing both *L. erythrorhizon* and *A. euchroma* cultured cells indicate the existence of a competing route branching out from the shikonin biosynthetic pathway to shikonofuran. A previous study has shown that the branch point is the transformation from (*Z*)-3’’-hydroxy-geranylhydroquinone to an aldehyde intermediate (*E*)-3’’-oxo-geranylhydroquinone. However, the gene encoding the oxidoreductase that catalyzes the branch reaction remains unidentified. In this study, we discovered a candidate gene belonging to the cinnamyl alcohol dehydrogenase family, *AeHGO*, through coexpression analysis of transcriptome data sets of shikonin-proficient and shikonin-deficient cell lines of *A. euchroma*. In biochemical assays, purified AeHGO protein reversibly oxidized (*Z*)-3’’-hydroxy-geranylhydroquinone to produce (*E*)-3’’-oxo-geranylhydroquinone followed by reversibly reducing (*E*)-3’’-oxo-geranylhydroquinone to (*E*)-3’’-hydroxy-geranylhydroquinone, resulting in an equilibrium mixture of the three compounds. Time course analysis and kinetic parameters showed that the reduction of (*E*)-3’’-oxo-geranylhydroquinone was stereoselective and efficient in presence of NADPH, which determined that the overall reaction proceeded from (*Z*)-3’’-hydroxy-geranylhydroquinone to (*E*)-3’’-hydroxy-geranylhydroquinone. Considering that there is a competition between the accumulation of shikonin and shikonofuran derivatives in cultured plant cells, AeHGO is supposed to play an important role in the metabolic regulation of the shikonin biosynthetic pathway. Characterization of AeHGO should help expedite the development of metabolic engineering and synthetic biology toward production of shikonin derivatives.

## Introduction

**Figure 1:**
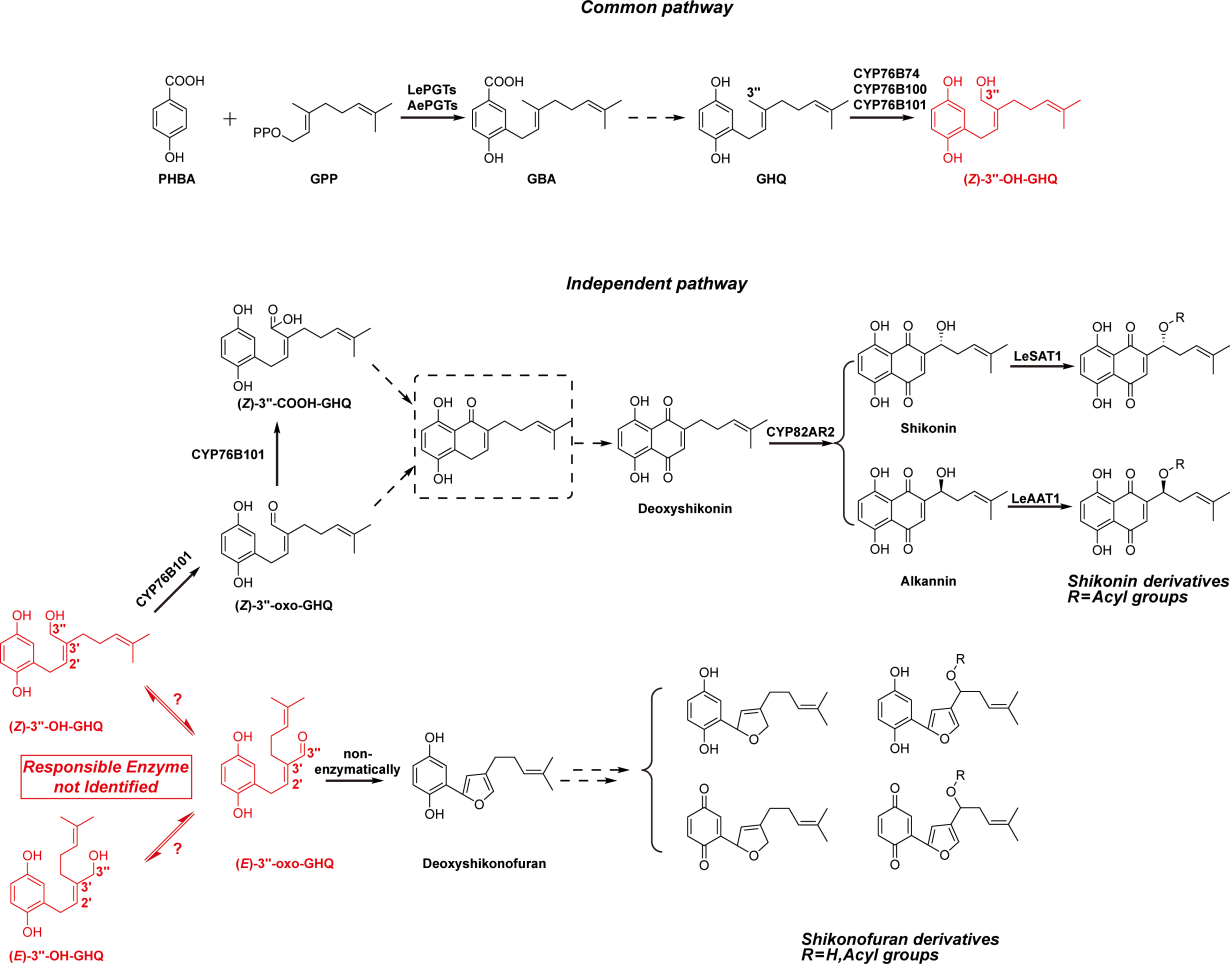
Simplified biosynthetic pathways of shikonin derivatives and shikonofuran derivatives. The branching reaction that directs the shikonin biosynthetic pathway to shikonofuran and the branch point intermediate (*Z*)-3’’-OH-GHQ are highlighted in red. The problem addressed in this study is highlighted in red box. Putative intermediates and yet unidentified reactions are denoted with dashed box and lines.

Shikonin and its derivatives are the main components of red pigment extracts from boraginaceous plants, including species belonging to the genera *Lithospermum*, *Arnebia*, *Anchusa*, *Alkanna*, *Echium*, and *Onosma* (Papageorgiou et al., 1999; Sun et al., 2022). These plants and their preparations have been used as natural dyes and herbal medicines in both Europe and the Orient for centuries, such as *Arnebiae Radix* used in traditional Chinese medicine (TCM) (Pharmacopoeia Committee of P. R. China, 2020). Shikonin derivatives are well recognized for their broad-spectrum activities against cancer, oxidative stress, bacteria, inflammation, and virus (Papageorgiou et al., 2006; Boulos et al., 2019). The naphthazarin (5, 8-dihydroxy-l, 4-naphthoquinone) pharmacophore in the chemical structures is the basis for the biological activity of shikonin derivatives (Wang et al., 2012). In addition, phytochemical studies on cultured cells of both *Lithospermum erythrorhizon* and *Arnebia euchroma* reveal the presence of a class of benzo/hydroquinones, shikonofuran derivatives, that accumulate competitively with shikonin derivatives (Yazaki et al., 1986; Yazaki et al., 1987; Fukui et al., 1992; Wang et al., 2014). This suggests a possible competing route branching out from the shikonin biosynthetic pathway into shikonofuran (**Figure 1**).

Understanding the biosynthetic process of the naphthoquinone ring in shikonin derivatives has posed a challenge for several decades. The metabolic regulation of the biosynthetic pathway remains largely unknown at the molecular level (Yazaki, 2017; Yadav et al., 2022). The shikonin biosynthetic pathway can be divided into two parts based on the location where the branch occurs: the common pathway and the independent pathway. For the common pathway, the first committed step is the condensation of *para*-hydroxybenzoic acid (PHBA) and geranyl diphosphate (GPP) catalyzed by *para*-hydroxybenzoic acid geranyltransferase (PGT), resulting in the formation of 3-geranyl-4-hydroxybenzoic acid (GBA) (Yazaki et al., 2002; Wang et al., 2023) (**Figure 1**). Then GBA is converted to geranylhydroquinone (GHQ) by an unknown mechanism. The subsequent oxidation at the C-3’’ position of GHQ is catalyzed by the CYPs of CYP76B subfamily: CYP76B74 and CYP76B100 catalyze the formation of (*Z*)-3’’-hydroxy-geranylhydroquinone [(*Z*)-3’’-OH-GHQ] (Wang et al., 2019; Song et al., 2020), and CYP76B101 catalyzes the production of a 3’’-carboxylic acid derivative of (*Z*)-3’’-OH-GHQ, i.e. (*Z*)-3’’-COOH-GHQ (Song et al., 2020) (**Figure 1**). *CYP76B74* RNA interference in *A. euchroma* hairy roots proves that (*Z*)-3’’-OH-GHQ is an intermediate of shikonin derivatives (Wang et al., 2019). (*Z*)-3’’-OH-GHQ is also considered to be a branch point of the shikonin biosynthetic pathway. After the formation of (*Z*)-3’’-OH-GHQ, the biosynthetic pathway divides into two branches, one leading to shikonin derivatives and the other to shikonofuran derivatives.

In the independent pathway leading to shikonin, it is speculated that the naphthoquinone ring comes from the cyclization of (*Z*)-3’’-oxo-GHQ or (*Z*)-3’’-COOH-GHQ (Yamamoto et al., 2000; Takanashi et al., 2019; Song et al., 2020; Tang et al., 2020). However, the cyclization product remains unidentified, and the reaction mechanism remains undetermined. The decoration enzymes such as CYP82AR2, LeSAT1, and LeAAT1 fulfill the transformation from deoxyshikonin to shikonin and its derivatives (**Figure 1**) (Oshikiri et al., 2020; Song et al., 2021). In the independent pathway branching out to shikonofuran, an alcohol dehydrogenase (ADH) activity, which catalyzes the branch reaction, has been characterized using partially purified crude enzyme of *L. erythrorhizon* cultured cells (Yamamoto et al., 2020). The activity involves the oxidation at 3’’-OH of (*Z*)-3’’-OH-GHQ to an aldehyde moiety concomitant with the isomerization at the C2’–C3’ double bond from the *Z*-form to the *E*-form. Spontaneous cyclization of the *E*-form product, (*E*)-3’’-oxo-geranylhydroquinone [(*E*)-3’’-oxo-GHQ], forms the characteristic furan ring of shikonofuran derivatives. However, the gene encoding the ADH that catalyzes the branch reaction is yet to be discovered. Its genetic information, reaction process and enzymatic characteristics remain enigmatic.

Here, we report the discovery and the characterization of a cinnamyl alcohol dehydrogenase (CAD)-like gene, *AeHGO*, encoding an oxidoreductase catalyzing the oxidation at 3’’-OH of (*Z*)-3’’-OH-GHQ and the isomerization at the C2’–C3’ double bond. The characterization of AeHGO has deepened the understanding of the branch mechanism in the shikonin biosynthetic pathway at the molecular level, which will provide additional layer of controlling large-scale shikonin production.

## Experimental procedures

### General experimental procedures

^1^H and ^13^C NMR spectra were recorded on Bruker DRX 500 spectrometer. The observed chemical shift values were reported in ppm. The UPLC and LC-MS analyses of the enzymatic products were performed as previously described with the exception that the solvent system comprised acetonitrile (A) and water (0.1% formic acid; B) at 0.5 mL min^-1^ with the following gradient program: 0 min, 30% A; 4 min, 70% A; 4.5 min, 100% A; 6.5 min, 100% A (Wang et al., 2019). A Waters ACQUITY UPLC-PDA system was equipped with an ACQUITY UPLC HSS T3 (2.1 × 50 mm, 1.8 *μ*m) column with an absorbance range of 210 to 400 nm. For the isolation of the enzymatic product, the same approach as previously described was employed (Wang et al., 2019).

### Plant materials, RNA sequencing and bioinformatic processing

The shikonin-proficient (SP) and shikonin-deficient (SD) suspension-cultured cell lines were derived from hypocotyls of *A. euchroma* and were grown in improved Linsmaier and Skoog liquid medium as described previously (Wang et al., 2014). The construction of transcriptome databases from the SP and SD suspension-cultured cell lines was previously reported (Wang et al., 2019).

### Chemicals

(*Z*/*E*)-3’’-hydroxy-geranylhydroquinone [(*Z*/*E*)-3’’-OH-GHQ] was chemically synthesized as described previously (Oshikiri et al., 2020). Cofactors FMN, NAD(P)^+^ and NAD(P)H were purchased from Solarbio (Beijing, China). Deuterium oxide and the substrates cinnamyl alcohol, *p*-coumaryl alcohol, coniferyl alcohol, sinapyl alcohol, and geraniol were purchased from Macklin (Shanghai, China).

### Isolation of cDNAs and cloning of candidate genes

Total RNA was extracted using TRIzol reagent (Invitrogen) and reverse-transcribed (RT) using a the PrimerScript First Strand cDNA Synthesis Kit (TaKaRa) with random primers and oligo(dT) at the same time. BLAST searches against unigenes of cluster 3 and cluster 6 in *A. euchroma* transcriptome database resulted in two candidate genes, *unigene18140* (*AeHGO*) and *unigene32185*, with sequence similarity to alcohol dehydrogenases (ADHs). Their full-length clones were acquired by PCR using the gene-specific primer pairs AeHGO-F1 (5′-TATGGAGCTCGGTACCATGGGAAATTCAGCAGAAC-3′) and AeHGO-R1 (5′- CGACAAGCTTGAATTCTTAGGCAGTTTTAAGAGTATTGC-3′), 32185-F1 (5′- TATGGAGCTCGGTACCATGTCCAACACTGCTGG-3′) and 32185-R1 (5′- CGACAAGCTTGAATTCTTAACCTTCCATGTTGATAATGC-3′). In these primers the extensions homologous to vector ends for subcloning were underlined. The PCR products were cloned into pEASY-Blunt Simple vector (TransGen) for sequencing and then subcloned into pCold II vector (Takara) using In-Fusion HD Cloning Kit (Takara), which resulted in the expression construct pCold II-*AeHGO* and pCold II-*unigene32185*.

### Heterologous expression in *E. coli* and purification of expressed AeHGO

The expression vectors pCold II-*AeHGO* and pCold II-*unigene32185* were introduced into the *E. coli* strain *Transetta* (TransGen) to produce a protein with a N-terminal His-tag. *E. coli* cultures carrying pCold II-*AeHGO* and pCold II-*unigene32185* were induced by adding 0.5 mM IPTG and grown at 15°C for 24 h. Cells containing recombinant protein were harvested by centrifugation, resuspended in either 5 mL of assay buffer (50 mM MES-Tris, pH 7.5, 5% glycerol) or in 5 mL of His-tag lysis buffer (20 mM NaH_2_PO_4_, pH 7.4, 500 mM NaCl, 10% glycerol, and 1 mM phenylmethanesulfonyl fluoride). The cells were disrupted by sonication and the lysates were cleared by centrifugation at 16,000 g (15 min). The resulting supernatants containing the soluble enzyme were either subjected to the enzymatic reaction or desalted on Amicon^®^ Ultra-15 Centrifugal Filter (10 K, Millipore) into loading buffer (20 mM NaH_2_PO_4_, pH 7.4, 500 mM NaCl, and 20 mM imidazole).

The supernatant of the bacterial lysate (5 mL) containing AeHGO protein was loaded onto a HisTrap™ FF crude column (1 mL, Cytiva) pre-equilibrated with loading buffer. After the sample was loaded, the column was washed with 20 mL of the loading buffer, followed by sequential elutions with 10 mL of the same buffer containing 50 mM, 100 mM, 200 mM, and 500 mM imidazole. The protein fractions were collected and desalted on Amicon^®^ Ultra-15 Centrifugal Filter into assay buffer. Protein purity was estimated by SDS-PAGE, followed by coomassie brilliant blue staining. The fraction with the highest degree of purity was used for further characterization. The protein concentration was determined by the Bradford method (Bradford, 1976).

### Oxidoreductase activity

For the quantitative determination of the oxidative activity, the basic reaction mixture (200 *µ*L) contained 50 mM MES-Tris (pH 7.5), 200 *µ*M (*Z*/*E*)-3’’-OH-GHQ, 500 *µ*M NAD(P)^+^ and 5 *µ*g of AeHGO protein. With regard to the reductive activity, the reaction mixture (200 *µ*L) contained 50 mM MES-Tris (pH 7.5), 200 *µ*M (*E*)-3’’-oxo-GHQ, 500 *µ*M NADPH and 5 *µ*g of AeHGO protein. The reaction mixtures were incubated at 37°C for 30 min, and the reactions were terminated by the addition of 600 *µ*L of acetonitrile. The protein was removed by centrifugation at 16,000 g for 20 min. The enzymatic products were analyzed by UPLC under the conditions described above. For the quantitative measurements of the enzyme activity, three parallel assays were carried out routinely.

### Biochemical properties of the recombinant enzymes

The assays (100 *µ*L) for the determinations of the kinetic parameters of the substrates contained 500 *µ*M NADP^+^ or NADPH, 170 ng of AeHGO protein, and substrates at final concentrations of 3 – 200 *µ*M. For the determination of the kinetic parameters of NADP^+^, (*Z*)-3’’-OH-GHQ at 500 *µ*M and NADP^+^ at final concentrations of 5 – 200 *µ*M were used. For each substrate or cofactor, the incubation time was controlled respectively so that the reaction was under 10% complete. Kinetic constants were calculated based on Michaelis-Menten kinetics using GraphPad Prism 8 (GraphPad Software).

To investigate the optimal pH, the enzyme reactions were performed in reaction buffers with pH values in the range of 6.5–8.0 (MES-Tris buffer), 7.5–9.5 (Tris-HCl buffer) at 37°C. To assay the optimal reaction temperature, the reaction mixtures were incubated at nine different temperatures that ranged from 0°C to 60°C in 50 mM MES-Tris buffer (pH 7.5). The effect of EDTA and divalent cations on the activity of AeHGO was investigated by addition of 500 *µ*M EDTA, MgCl_2_, CaCl_2_, MnCl_2_, FeCl_2_, CoCl_2_, NiCl_2_, CuCl_2_ or ZnCl_2_ respectively with (*Z*)-3’’-OH-GHQ as the substrate.

### Preparative synthesis of the enzymatic products for structural elucidation

The assay for the isolation of the enzymatic product (*E*)-3’’-oxo-GHQ (**3**) (25 ml) contained 50 mM MES-Tris (pH 7.5), 500 *µ*M (*Z*)-3’’-OH-GHQ (**1**), 1 mM NADP^+^ and 3 mg AeHGO protein. The reaction mixture was incubated at 37°C for 4 h and subsequently extracted with ethyl acetate (30 ml × 3). After evaporation of the solvent, the residues were dissolved in methanol and purified by reverse-phase semi-preparative HPLC under the conditions described above. The isolated product was subjected to MS and ^1^H and ^13^C NMR spectroscopic analyses, which yielded the following results.

(*E*)-3’’-oxo-GHQ (**3**): TOF-MS, *m*/*z*: 259.1 [M-H]^-^; ^1^H NMR (500 MHz, acetone-*d*_6_): *δ* 9.42 (1H, *s*, CHO), 6.72 (1H, *d*, *J* = 8.5 Hz, H-6), 6.70 (1H, *t*, *J* = 7.5 Hz, H-2’), 6.65 (1H, *d*, *J* = 3 Hz, H-3), 6.57 (1H, *dd*, *J* = 8.5, 3 Hz, H-5), 5.18 (1H, br. *t*, *J* = 7.5, 1 Hz, H-6’), 3.65 (2H, *d*, *J* = 7.5 Hz, H-1’), 2.38 (2H, *t*, *J* = 7.5 Hz, H-4’), 2.09 (2H, br. *q*, *J* = 7.5 Hz, H-5’), 1.66 (3H, br. *s*, H-7’’), 1.59 (3H, br. *s*, H-8’) (**Figure S5**); ^13^C NMR (125 MHz, acetone-*d*_6_),: *δ* 194.49 (CHO), 147.87 (C-1), 125.66 (C-2), 116.54 (C-3), 150.56 (C-4), 113.89 (C-5), 115.71 (C-6), 152.90 (C-2’), 142.89 (C-3’), 23.83 (C-4’), 26.99 (C-5’), 123.76 (C-6’), 131.73 (C-7’), 24.93 (C-8’), 16.82 (C-7’’) (**Figure S6**).

### *In silico* sequence analysis

The unigenes were clustered using the fuzzy c-means algorithm Mfuzz. The encoded polypeptides of *AeHGO* and *unigene32185*, reported CADs from *Arabidopsis thaliana*, geraniol dehydrogenase (GEGH) from *Ocimum basilicum* and 10-hydroxygeraniol oxidoreductases (10HGO) from *Catharanthus roseus* were aligned using ClustalW (Larkin et al., 2007). Phylogenetic analyses were conducted using MEGA-X (Kumar et al., 2018). Protein sequences were aligned using the MUSCLE program (Edgar, 2004). Phylogenetic relationships were reconstructed by the maximum likelihood method based on the JTT/+G model (five categories) and a bootstrap of 1,000 replicates. Bootstrap values were indicated in percentages (only those >65% were presented) on the nodes. The bootstrap values were obtained from 1000 bootstrap replicates. The scale bar corresponded to 0.2 estimated amino acid changes per site.

## Results

### The screening of the gene(s) responsible for dehydrogenation of (*Z*)-3’’-OH-GHQ

Due to absence of the coding gene sequence(s) of the enzyme(s) responsible for the branch reaction, we mined the candidate gene(s) using the transcriptome data sets obtained from the red shikonin-proficient (SP) cell line, the white shikonin-deficient (SD) cell line, and the SD cells treated with a time-series of MeJA elicitation (Wang et al., 2014; Wang et al., 2019). A stepwise screening strategy was employed based on the following three criteria: firstly, the expression levels of candidate gene(s) should be comparable to those of functional genes in the shikonin biosynthetic pathway, such as *AePGT*s and *CYP76B74*; secondly, the expression patterns of the candidate gene(s) should be consistent with those of functional genes in the shikonin biosynthetic pathway and precursor pathways, i.e. the mevalonic acid (MVA) and phenylpropanoid pathways; thirdly, the candidate gene(s) should be predicted as an ADH-like gene which has a NAD(P)^+^ binding domain in the protein structure.

**Figure 2:**
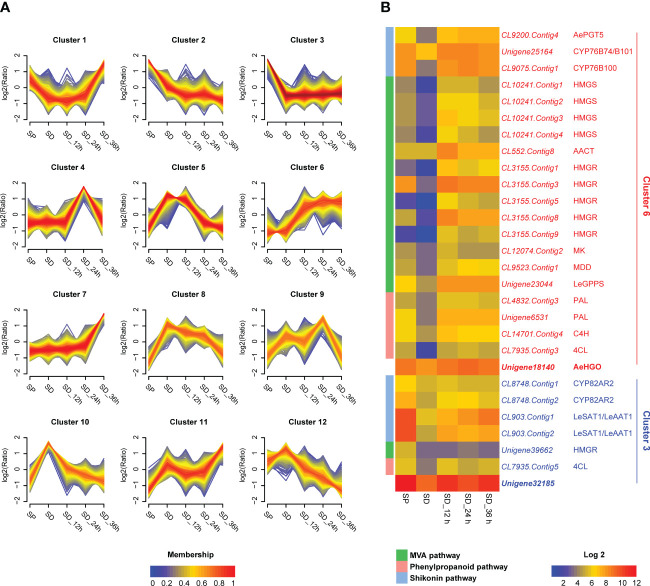
Transcriptome analysis of unigenes in SP cell, SD cell, and SD cells in different MeJA elicitation times. **(A)** Clustering results of the unigenes according to temporal expression patterns in different cell lines (7637 unigenes analyzed). The expression level changes are indicated as log2 fold change. FPKM (Fragments Per Kilobase Million) values were directly used to calculate clustering of unigenes using the fuzzy c-means algorithm Mfuzz (Kumar and Futschik, 2007) with optimal fuzzifier “m” value and “min.acore” value of 0.7. Membership values are color-encoded with red shades denoting high membership values and blue shades denoting low membership values of unigenes. **(B)** Expression patterns of candidate genes as well as mevalonic acid (MVA), phenylpropanoid, and shikonin pathway genes. The log2 transformed FPKM values are used for preparing the heatmaps. Abbreviations: SP, shikonin-proficient cell line; SD, shikonin-deficient cell line; SD_12 h/24 h/36 h, shikonin-deficient cell line after treated with MeJA for 12 h/24 h/36 h; AePGT5, *A. euchroma para*-hydroxybenzoic acid geranyltransferase 5; HMGS, 3-hydroxy-3-methylglutaryl-CoA synthase; AACT, acetoacetyl-CoA thiolase; HMGR, 3-hydroxy-3-methylglutaryl-CoA reductase; MK, mevalonate kinase; MDD, mevalonate diphosphate decarboxylase; LeGPPS, *L. erythrorhizon* geranyl diphosphate synthase; PAL, L-phenylalanine ammonia lyase; C4H, cinnamate 4-hydroxylase; 4CL, 4-coumarate CoA-ligase; LeSAT1, *L. erythrorhizon* shikonin O-acyltransferase 1; LeAAT1, *L. erythrorhizon* alkannin O-acyltransferase 1.

Specifically, in the first step of screening, the unigenes with a FPKM value less than 30 were filtered out. As a result, 7637 of the total 121239 unigenes were retained and used as input for the next round of screening (**Table S1**). In the second step of screening, the 7637 unigenes were grouped into 12 clusters according to their temporal expression patterns using Mfuzz (Kumar and Futschik, 2007) (**Figure 2A**; **Table S2**). The unigenes in the common pathway of shikonin biosynthesis, MVA and phenylpropanoid pathways were mainly grouped into cluster 6 (**Figures 1**, **2B**). For unigenes in cluster 6 (639 in total), the transcriptional level in the SP cell line was higher than in the SD cell line without MeJA elicitation treatment (**Figure 2**). The transcriptional level of these unigenes increased significantly after MeJA elicitation, and the value peaked after 12-24 hours in the SD cell line (**Figure 2**). This expression pattern was consistent with the accumulation pattern of shikonofuran derivatives in the SD cell line under MeJA elicitation (Wang et al., 2014). By contrast, enzymes in the independent pathway leading to shikonin derivatives, such as homologs of CYP82AR2, LeSAT1, and LeAAT1 in *A. euchroma*, were grouped in cluster 3 (**Figures 1**, **2B**). The transcriptional level of cluster 3 unigenes (1106 in total) in the SP cell line was always higher than in the SD cell line regardless of MeJA elicitation (**Figure 2**). The unigenes in cluster 3 and cluster 6 were eventually used as input for the next round of screening. In the third step of screening, ADH sequences from *Zea mays* (GenBank accession no. P00333.1), *Arabidopsis thaliana* (GenBank accession no. NP_188576.1), and *Pinus banksiana* (GenBank accession no. AAC49540.1) were employed as queries to retrieve the unigenes in cluster 3 and cluster 6 using BLAST+ program. As a result, two candidate genes, *unigene18140* and *unigene32185*, were selected from cluster 6 and cluster 3 respectively for further analysis (**Figure 2B**).

**Figure 3:**
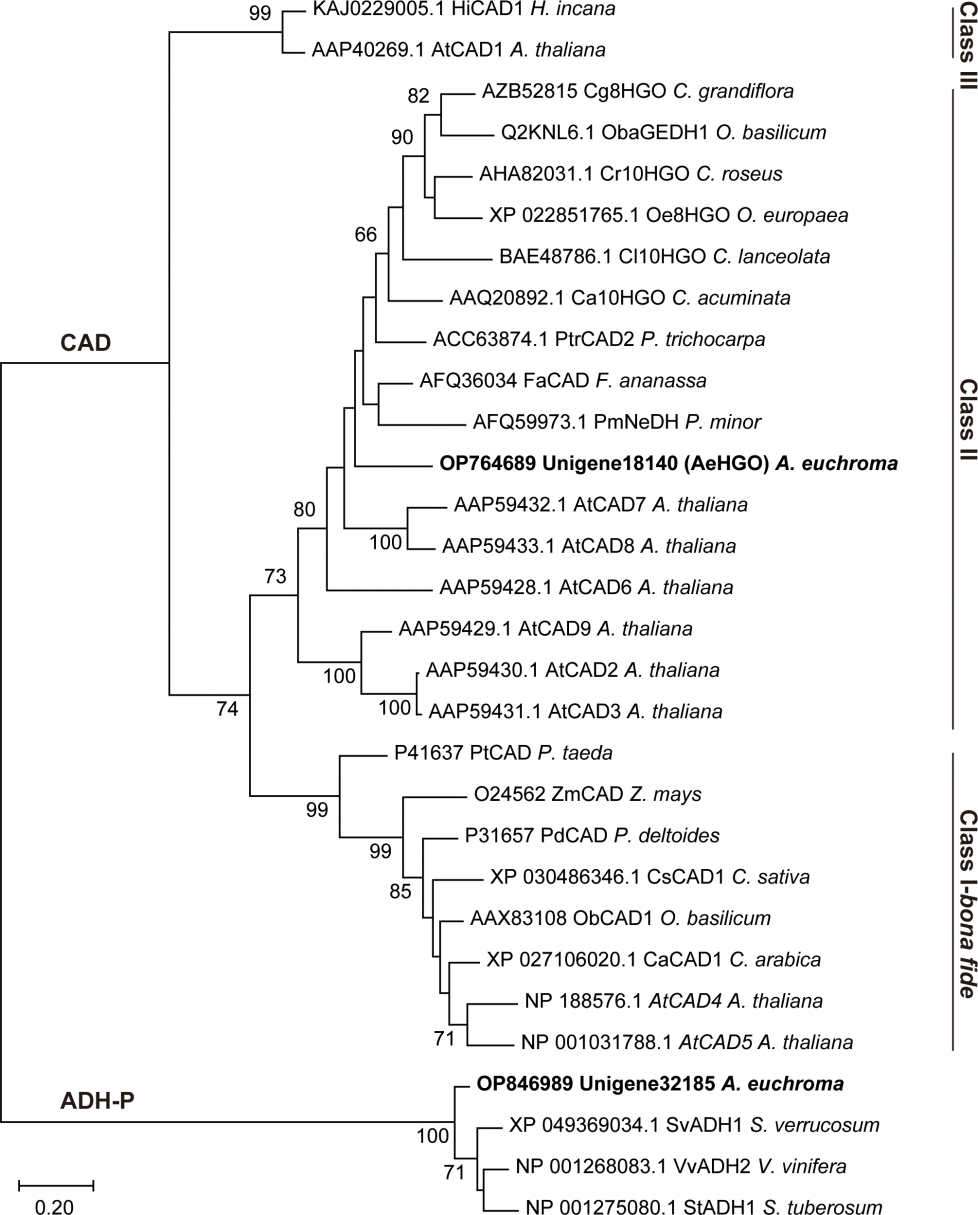
Maximum likelihood phylogeny of the unigene18140 and unigene32185 proteins and the related plant ADHs. Nodes with bootstrap values >65 are indicated by numbers on the branch.

The results of a multiple alignment and homology research showed that *unigene18140* had high homology with members of cinnamyl alcohol dehydrogenase (CAD) family and had a high identity to 10-hydroxygeraniol oxidoreductases (10HGO) (**Figure S1**) (Kim et al., 2004; Krithika et al., 2015). *Unigene32185* belonged to the plant alcohol dehydrogenase (ADH-P) family (Strommer, 2011). Consistently, the phylogenetic analysis clustered *unigene18140* into class II of the CAD family and *unigene32185* into the ADH-P family (**Figure 3**). The members in class II of the CAD family are featured with a more broad substrate spectrum than class I members, which are highly conserved in substrate specificity and associated with primary lignin synthesis (Preisner et al., 2018). The dehydrogenases using (hydroxy)geraniol as the substrate also fell into the same class with unigene18140, indicating that unigene18140 may be involved in the oxidation of hydroxy geranyl side chain of (*Z*)-3’’-OH-GHQ (Iijima et al., 2006; Krithika et al., 2015). Members of ADH-P family catalyze the interconversion between alcohol and acetaldehyde, and therefore play a role in the anaerobic response (Strommer, 2011).

The two candidates were heterologously expressed in *E*. *coli*, and the resultant crude protein extracts were tested for the reactivity with (*Z*)-3’’-OH-GHQ (**1**). As a result, the candidate unigene18140, which was named as AeHGO [*A. euchroma* (*Z*)-3’’-hydroxy-geranylhydroquinone oxidoreductase], was found to catalyze the conversion of (*Z*)-3’’-OH-GHQ (**1**) in the presence of NAD(P)^+^ (**Figure S2**). Due to the lack of activity when using (*Z*)-3’’-OH-GHQ (**1**) as a substrate, the ADH-P-like candidate gene *unigene32185* was not investigated further.

### *In vitro* enzyme activity assays for AeHGO

**Figure 4:**
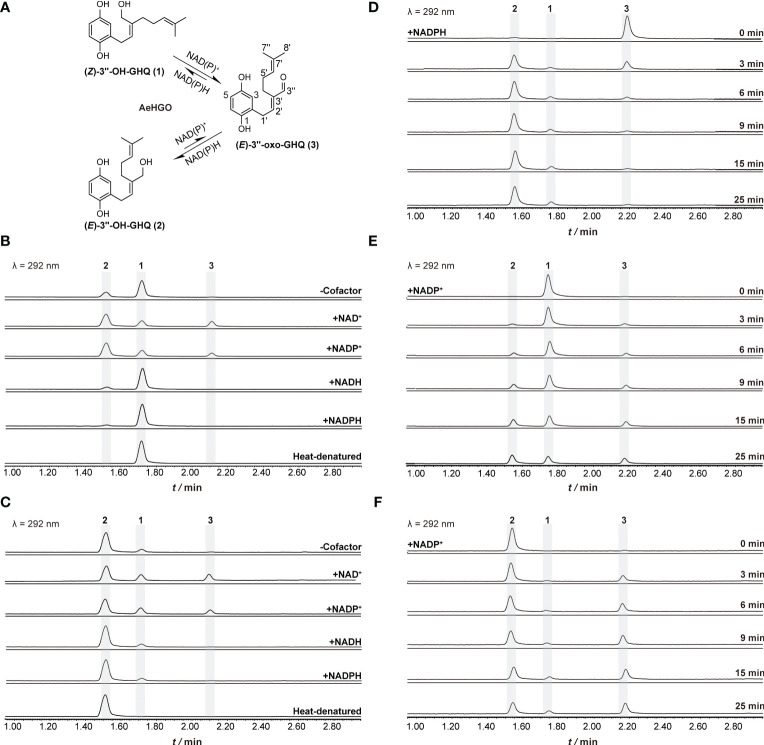
Functional characterization of recombinant AeHGO and time-course analysis of the enzymatic reactions. The detection wavelength is set at 292 nm. The reaction mixture containing heat-denatured AeHGO is used as a control. **(A)** Process of the reaction catalyzed by recombinant AeHGO. The chemical structures of the compounds and corresponding peaks in UPLC chromatogram are marked as 1–3. The length of an arrow represents relative reaction velocity. **(B)** Chromatograms showing reactions containing (*Z*)-3’’-OH-GHQ (1) with AeHGO protein in the presence or absence of cofactors. **(C)** Chromatograms showing reactions containing (*E*)-3’’-OH-GHQ (2) with AeHGO protein in the presence or absence of cofactors. **(D)** Chromatograms showing reactions using (*E*)-3’’-oxo-GHQ (3) as the substrate with NADPH for 0–25 min. **(E)** Chromatograms showing reactions using (*Z*)-3’’-OH-GHQ (1) as the substrate with NADP^+^ for 0–25 min. **(F)** Chromatograms showing reactions using (*E*)-3’’-OH-GHQ (2) as the substrate with NADP^+^ for 0–25 min.

The AeHGO recombinant protein was purified *via* immobilized metal ion affinity chromatography (IMAC). About 1.0 mg/mL of recombinant AeHGO was acquired with sufficient purity (**Figure S3**). The oxidative activity of purified AeHGO was characterized concerning different substrates and cofactor specificities (**Figure 4A**). When (*Z*)-3’’-OH-GHQ (**1**) was employed as the substrate, (*E*)-3’’-OH-GHQ (**2**) and an unknown product (**3**) appeared in the reaction system with both the oxidized coenzyme NAD^+^ and NADP^+^ (**Figure 4B**). When the coenzyme was not present or the reduced coenzyme NAD(P)H was present, the unknown product (**3**) disappeared and only the isomer (*E*)-3’’-OH-GHQ (**2**) was observed. A similar result occurred with (*E*)-3’’-OH-GHQ (**2**) as the substrate. Only the isomerization reaction was observed without cofactor or with reduced coenzymes, and the addition of the oxidized coenzymes led to the unknown product (**3**) (**Figure 4C**). These results suggested that AeHGO possessed the isomerization activity towards (*Z*/*E*)-3’’-OH-GHQ (**1**/**2**), and the unknown product (**3**) represented most likely an oxidized product.

The enzymatic product (**3**) was subsequently purified and analyzed by MS and NMR. The NMR data (**Figures S5**, **S6**) of this compound was in good agreement with those of (*E*)-3’’-oxo-GHQ (**3**) (Yamamoto et al., 2020). Therefore, this enzymatic product was unambiguously identified as (*E*)-3’’-oxo-GHQ (**3**), which indicated that AeHGO was able to catalyze the branch reaction toward shikonofuran derivatives.

### Time-course and kinetic analysis of the reactions catalyzed by AeHGO

To further study the enzymatic reaction process, time-course experiments were conducted with (*Z*)-3’’-OH-GHQ (**1**), (*E*)-3’’-OH-GHQ (**2**) and (*E*)-3’’-oxo-GHQ (**3**) as the substrates (**Figure 4A**). When (*E*)-3’’-oxo-GHQ (**3**) was employed as the substrate in the presence of NADPH, the reductive reaction was found to be stereoselective. (*E*)-3’’-oxo-GHQ (**3**) was almost completely consumed in 10 min and the yield of (*E*)-3’’-OH-GHQ (**2)** was much higher than (*Z*)-3’’-OH-GHQ (**1**) (**Figure 4D**). The result led to an inference that AeHGO tend to transform (*Z*)-3’’-OH-GHQ (**1**) to (*E*)-3’’-OH-GHQ (**2**) through (*E*)-3’’-oxo-GHQ (**3**) (**Figure 4A**). This inference was confirmed when (*Z*)-3’’-OH-GHQ (**1**) and (*E*)-3’’-OH-GHQ (**2**) were used as substrates. At the initial phase of the reaction with (*Z*)-3’’-OH-GHQ (**1**) in the presence of NADP^+^, the enzymatic products (*E*)-3’’-OH-GHQ (**2**) and (*E*)-3’’-oxo-GHQ (**3**) appeared nearly at the same time (**Figure 4E**). As the reaction continued, the accumulation of (*E*)-3’’-OH-GHQ (**2**) was faster than its oxidized product (*E*)-3’’-oxo-GHQ (**3**). When the reaction finished at 25 min, (*E*)-3’’-OH-GHQ (**2**) accounted for about 40% of the reaction mixture, which was higher than (*Z*)-3’’-OH-GHQ (**1**) (33%) and (*E*)-3’’-oxo-GHQ (**3**) (27%) (**Figure 4E**). By contrast, when (*E*)-3’’-OH-GHQ (**2**) was employed as the substrate in the presence of NADP^+^, the accumulation of (*Z*)-3’’-OH-GHQ (**1**) was much slower than (*E*)-3’’-oxo-GHQ (**3**) (**Figure 4F**). The higher accumulation of (*E*)-3’’-oxo-GHQ (**3**) was caused by the inefficiency of transformation from (*E*)-3’’-oxo-GHQ (**3**) to (*Z*)-3’’-OH-GHQ (**1**) (**Figure 4A**).

To further understand the aforementioned enzymatic behaviors, we evaluated different kinetic parameters of AeHGO (**Table 1**). The apparent *k*_cat_ value for (*Z*)-3’’-OH-GHQ (**1**) was comparable to NADP^+^ and about twice as for its *E*-form (**2**) at saturated concentration of NADP^+^. Similar trend was observed concerning the $k_{cat}K_{m}^{-1}$ values between these three substrates. These results showed that the *Z*-form (**1**) was dehydrogenated by AeHGO more effciently than the *E*-form (**2**), consistent with the overall transformation from the *Z*-form (**1**) to the *E*-form (**2**). In the reductive reaction, the apparent *K*_m_ value for (*E*)-3’’-oxo-GHQ (**3**) was much lower than for (*Z*)-3’’-OH-GHQ (**1**), (*E*)-3’’-OH-GHQ (**2**), and NADP^+^. Together with the higher $k_{cat}K_{m}^{-1}$ value in the reductive reaction, the result suggested that the reduction catalyzed by AeHGO was more efficient than oxidation, which accelerated the transformation from (*Z*)-3’’-OH-GHQ (**1**) to (*E*)-3’’-OH-GHQ (**2**) (**Figure 4A**). Considering the reversibility of the reaction catalyzed by AeHGO, the stereoselective reduction of (*E*)-3’’-oxo-GHQ (**3**) to (*E*)-3’’-OH-GHQ (**2**) may have caused a higher dehydrogenation rate of (*Z*)-3’’-OH-GHQ (**1**) than (*E*)-3’’-OH-GHQ (**2**) (**Figure 4A**).

**Table 1:** Kinetic parameters of AeHGO.

Substrate	*K*_m_ (*µ*M)	*V*_max_ (pkat/*µ*g protein)	*k*_cat_ (s^-1^)	$k_{cat}K_{m}^{-1}$ (*µ*M^-1^·s^-1^)
(*Z*)-3’’-OH-GHQ (**1**)	24.68 ± 5.18	63.47 ± 3.79	2.58 ± 0.15	0.10
(*E*)-3’’-OH-GHQ (**2**)	18.70 ± 5.39	33.95 ± 2.28	1.38 ± 0.09	0.07
NADP^+^	17.81 ± 5.62	56.01 ± 3.99	2.28 ± 0.16	0.13
(*E*)-3’’-oxo-GHQ (**3**)	9.96 ± 2.87	69.79 ± 9.33	2.84 ± 0.38	0.28

Consistent with previous reports on CADs reaction kinetics (Bomati and Noel, 2005), a substrate inhibition phenomenon was observed when kinetic parameters of reductive reaction were measured (**Figure S9B**). However, detection of the substrate inhibition constant *K*_i_ failed, perhaps due to tight coupling of *K*_i_ and *K*_m_ (Eszes et al., 1996). The kinetic parameters of reductive reaction were measured at low substrate concentrations (**Figure S9A**). Within the concentration range applied here, the reductive reaction nearly obeyed Michaelis-Menten kinetics.

### Biochemical properties and substrate specificities of the recombinant AeHGO

**Figure 5:**
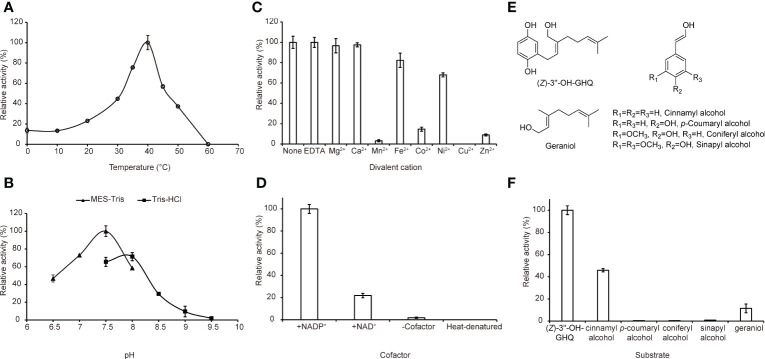
Biochemical properties of AeHGO. For the quantitative measurements of the enzyme activity, three parallel assays are carried out. The error bar represents the standard deviation of a measurement. For each quantitative measurement, relative activity is shown with the sample having the highest activity set arbitrarily at 100%. **(A)** Effects of temperature on the enzyme activities of AeHGO. **(B)** pH dependences of AeHGO. **(C)** Effects of various divalent metal ions on AeHGO activities. **(D)** Cofactor dependences of AeHGO. **(E)** Chemical structures of used substrates in specificity analysis. **(F)** Comparison of substrate specificities of purified AeHGO.

Previous studies have reported that the plant CAD activity was dependent on divalent cations and strongly affected by pH and temperature. Further investigations using (*Z*)-3’’-OH-GHQ (**1**) as the substrate and NADP^+^ as the cofactor in this study revealed that the highest activities occurred at about 37°C and the activity decreased rapidly at temperatures above 40°C (**Figure 5A**). The analysis of the enzyme activity within the pH range of 6.5 to 9.5 revealed that the optimal pH value was about 7.5 (**Figure 5B**). The addition of 0.5 mM Mg^2+^, Ca^2+^, and EDTA had no significant effect on the activity in comparison to the reaction with no ions. The involvement of Fe^2+^ and Ni^2+^ reduced the activity slightly. The reaction was severely hampered by Mn^2+^, Co^2+^, and Zn^2+^, and totally terminated by Cu^2+^ (**Figure 5C**). According to previous reports, the reason for inactivation caused by Zn^2+^ treatment is likely the oligomerization of CADs. EDTA treatment could prevent this process (Pandey et al., 2014). As illustrated in **Figure 5D**, NADP^+^ was the most favorable cofactor of AeHGO when using (*Z*)-3’’-OH-GHQ (**1**) as the substrate, with a *K*m value of 17.81 ± 5.62 *µ*M and a *k*_cat_ value of 2.28 ± 0.16 s^-1^. This result was consistent with the multiple sequence alignment of AeHGO and ADHs, which identified a conserved Ser216 involved in determining cofactor specificity (**Figure S1**). The side-chain of Ser216 forms a hydrogen bond with the 2’-phosphate group of NADP(H) and enables a preference for NADP(H) over NAD(H) (Youn et al., 2006). To further examine substrate specificity of AeHGO, a number of cinnamyl alcohol derivatives and geraniol were tested (**Figures 5E, F**). (*Z*)-3’’-OH-GHQ (**1**) was the optimal substrate of AeHGO. For the other substrates, cinnamyl alcohol and geraniol were dehydrogenated by AeHGO more efficiently. *p*-coumaryl alcohol, coniferyl alcohol and sinapyl alcohol, which are phenylpropanoid intermediates in the lignin biosynthetic pathway, were poorly recognized by AeHGO (Vanholme et al., 2019). This is consistent with the observation that AeHGO is clustered into class II of the CAD family together with (hydroxy)geraniol dehydrogenases, which is distinguished from the *bona fide* CADs by the substrate spectrum (**Figure 3**).

## Discussion

Previous studies revealed that shikonin derivatives and shikonofuran derivatives shared a common biosynthetic route branching out from (*Z*)-3’’-OH-GHQ (**1**). It was presumed that direct oxidation of (*Z*)-3’’-OH-GHQ (**1**) to (*Z*)-3’’-oxo-GHQ could easily lead to a C-C bond with the aromatic nucleus to form the naphthoquinone ring by an electrophilic reaction (Yamamoto et al., 2000). In contrast to that, (*E*)-3’’-oxo-GHQ (**3**) was found to be converted to deoxyshikonofuran, a benzo/hydroquinone metabolite rather than naphthoquinone (Yamamoto et al., 2020). It was speculated that the distance between the aromatic ring and the aldehyde group of (*E*)-3’’-oxo-GHQ (**3**) was disadvantageous for naphthoquinone ring formation (Yamamoto et al., 2020). Hence the configuration change of C2’–C3’ double bond from *Z* to *E* triggered by the oxidation of C3’’ shifts the metabolic flux from biosynthesis of shikonin derivatives to shikonofuran derivatives. In the present work, through an initial bioinformatics screen of the transcriptome data of *A. euchroma* for a potential (*Z*)-3’’-OH-GHQ (**1**) dehydrogenase, two candidates numbered *unigene18140* and *unigene32185* were identified. The *in vitro* activities of the encoded proteins were subsequently examined through heterologous expression in *E. coli*. Unigene18140 turned out to be an oxidoreductase reversibly catalyzing the oxidation of the 3’’-alcoholic group of (*Z*/*E*)-3’’-OH-GHQ (**1**/**2**) and the isomerization at the C2’–C3’ double bond. The main outcome of the reaction was transforming (*Z*)-3’’-OH-GHQ (**1**) to (*E*)-3’’-OH-GHQ (**2**) *via* (*E*)-3’’-oxo-GHQ (**3**). In other words, the products with the *E* configuration of C2’–C3’ double bond accounted for a large proportion in the equilibrium mixture. This enzyme was named AeHGO, and it was supposed to switch the shikonin biosynthetic pathway to shikonofuran. The inducible expression of *AeHGO* by MeJA was in consistent with the inducible accumulation of shikonofuran in SD cell lines, which provided further evidence for the function of AeHGO. Through homology research toward the genome sequence of *L. erythrorizon* using *AeHGO* as the query, Leryth_023241 (GenBank accession no. KAG9149627.1) of 95% identity was acquired (Auber et al., 2020). The coding gene of Leryth_023241 was significantly overexpressed in whole root tissue relative to leaf/stem tissue, and in periderm tissue relative to vascular tissues (Suttiyut et al., 2022). These results were consistent with the presence of shikonofuran derivatives in *L. erythrorizon*.

It is worth noting that the equilibrium state of the reaction catalyzed by AeHGO is different to the results obtained from *L. erythrorhizon*. As observed in *L. erythrorhizon*, (*Z*)-3’’-OH-GHQ (**1**) was mainly transformed to (*E*)-3’’-oxo-GHQ (**3**) rather than (*E*)-3’’-OH-GHQ (**2**) (Yamamoto et al., 2020). In the present SD cell lines of *A. euchroma*, the homologous genes of AeHGO retrieved from the transcriptome database were all expressed at a much lower level compared to AeHGO (**Table S3**). Therefore, the existence of a AeHGO homolog responsible for the accumulation of (*E*)-3’’-oxo-GHQ (**3**) in the present cell lines was ruled out. Coincidentally, when AeHGO crude enzyme extracted from *E. coli* was tested for the reactivity with (*Z*)-3’’-OH-GHQ (**1**), (*E*)-3’’-oxo-GHQ (**3**) also appeared as the main product (**Figure S2**). A hypothesis could be proposed that protein interactions may influence the catalytic behavior of AeHGO both in the plant cell lines and *E. coli* crude enzyme.

**Figure 6:**
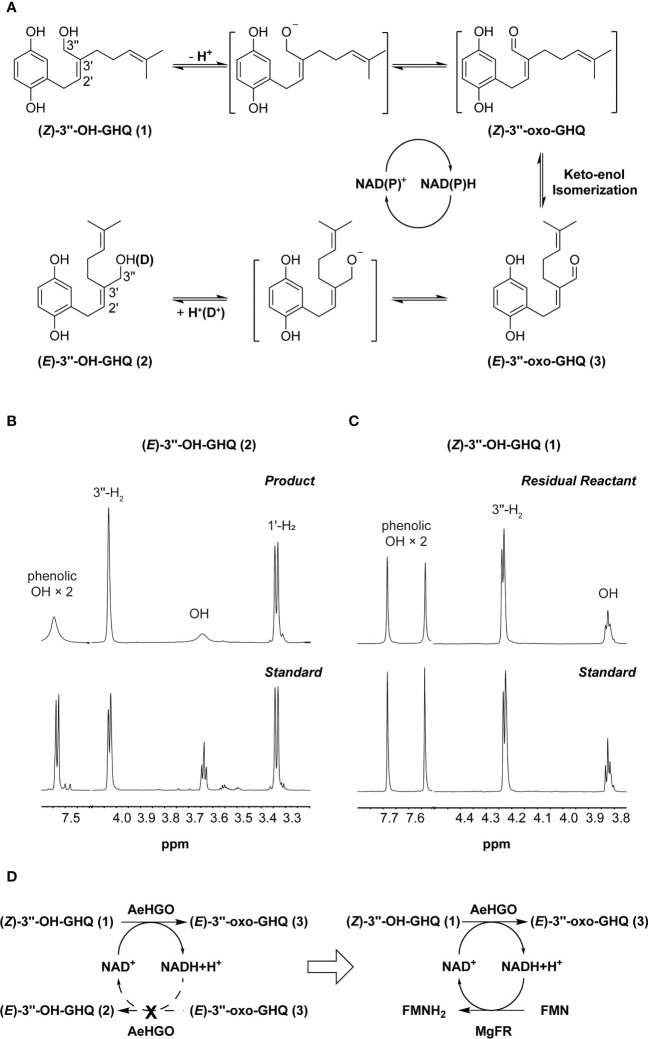
**A** proposed mechanism for reaction catalyzed by AeHGO. **(A)** Schematic diagram of AeHGO-catalyzed reversible reaction. (*Z*)-3’’-oxo-GHQ and the other transition states are shown inside the brackets. Hydrogen/deuterium exchange is shown inside parentheses. **(B)** Changes in ^1^H spectrum pattern of phenolic hydroxyls, alcoholic hydroxyl, 1’-H_2_, and 3’’-H_2_ of the enzymatic product **(*E*)**-3’’-OH-GHQ (**2**) after the reaction in D_2_O. Chemically synthesized **(*E*)**-3’’-OH-GHQ (**2**) was used as a control. **(C)** Changes in ^1^H spectrum pattern of phenolic hydroxyls, alcoholic hydroxyl, and 3’’-H_2_ of the residual reactant (*Z*)-3’’-OH-GHQ (**1**) after the reaction in D_2_O. Chemically synthesized (*Z*)-3’’-OH-GHQ (**1**) is used as a control. **(D)** Symbolic graph displaying the introduction of a competitive pathway. Dashed arrows signify the original pathway which is replaced by the artificially engineered competitive route.

As noted earlier, the configuration change of C2’–C3’ double bond from *Z* to *E* was triggered by the oxidation of C3’’. Accordingly, the reaction mechanism can be proposed that (*Z*/*E*)-3’’-OH-GHQ (**1**/**2**) was firstly dehydrogenated to form (*Z*/*E*)-3’’-oxo-GHQ. Then the keto–enol tautomerization of (*Z*/*E*)-3’’-oxo-GHQ afforded a facile rotation of the C2’–C3’ bond, which resulted in the configuration switching (**Figure 6A**). To obtain details of the reaction mechanism, the reaction solvent was replaced with deuterium oxide (D_2_O), and (*Z*)-3’’-OH-GHQ (**1**) was used as the substrate in the presence of NADP^+^. After the reaction, the enzymatic product (*E*)-3’’-OH-GHQ (**2**) and residual reactant (*Z*)-3’’-OH-GHQ (**1**) were purified and analyzed by ^1^H NMR. As shown in **Figure 6B**, compared with the standard, the triplet signal of OH diminished to a trace in the product (*E*)-3’’-OH-GHQ (**2**) (**Figures S7**, **S8**). And the doublet signal of 3’’-H_2_ in the standard became a broaden singlet signal (**Figure 6B**). As a comparison, the signals in the residual reactant (*Z*)-3’’-OH-GHQ (**1**) were only slightly altered due to hydrogen/deuterium exchange in D_2_O (**Figure 6C**). Taken together, these results indicated that the hydroxyl hydrogen in the substrate (*Z*)-3’’-OH-GHQ (**1**) was replaced by deuterium in the product (*E*)-3’’-OH-GHQ (**2**) (**Figure 6A**). With reference to the reaction mechanism of ADHs (Dołęga, 2010; Plapp, 2010; Raj et al., 2014), the AeHGO reaction was presumed to start with the dissociation of a proton from the alcoholic hydroxyl group of (*Z*)-3’’-OH-GHQ (**1**) (**Figure 6A**). The removal of hydrogen from the adjoining C-3’’ as a hydride (H^-^) followed closely. The hydride was transferred to NAD(P)^+^, and the reduced NAD(P)H was used in the reduction of (*E*)-3’’-oxo-GHQ (**3**). The addition of a proton/deuterium positive ion to the alkoxide ion ended the reaction. Moreover, the phenolic hydroxyl groups also underwent rapid hydrogen/deuterium exchange during the reaction.

It could be drawn that the transformation from (*Z*)-3’’-OH-GHQ (**1**) to (*E*)-3’’-OH-GHQ (**2**) *via* (*E*)-3’’-oxo-GHQ (**3**) was accelerated by the NAD(P)^+^ recycle (**Figure 6A**). The efficient stereoselective reduction from (*E*)-3’’-oxo-GHQ (**3**) to (*E*)-3’’-OH-GHQ (**2**) is the prerequisite of the NAD(P)^+^ recycle (**Figure 4D**). On the other hand, if the NAD(P)^+^ recycle is disturbed, the equilibrium between the three compounds may be broken. In order to verify this hypothesis, we introduced an enzyme which competed with AeHGO for oxidizing the newly formed NAD(P)H (**Figure 6D**). For this purpose, MgFR, a flavin oxidoreductase from *Mycobacterium goodii* X7B catalyzing the reduction of free flavins using NADH, was tested in the following study (Li et al., 2005; Chen et al., 2019). MgFR was heterologously expressed in *E. coli* and acquired in sufficient purity (**Figure S4**). Then the purified MgFR protein was added to the AeHGO reaction system together with FMN and NAD^+^ as cofactors. Contrary to expectations, the presence of MgFR did not change the contents of equilibrium mixture. This suggests that the reduced cofactor NAD(P)H is not released from the active center of AeHGO, but used immediately in the recycling. The same explanation could apply for the undetected intermediate (*Z*)-3’’-oxo-GHQ, which is transformed immediately after its formation in the active center. As the putative intermediate of shikonin derivatives, (*Z*)-3’’-oxo-GHQ may be produced by another unknown dehydrogenase. Considering the instability of (*Z*)-3’’-oxo-GHQ (Yamamoto et al., 2020), the dehydrogenase may exist as a part of a metabolon, allowing instant transformation of the unstable intermediate to the final product (Stefely and Pagliarini, 2017).

In summary, AeHGO, the key enzyme which controlled the branching of shikonin biosynthetic pathway was identified. Through transforming (*Z*)-3’’-OH-GHQ (**1**) to (*E*)-3’’-OH-GHQ (**2**) *via* (*E*)-3’’-oxo-GHQ (**3**), it led the metabolic flux to the biosynthesis of shikonofuran derivatives. These results clarified the branch mechanism in the shikonin biosynthetic pathway at the molecular level, which should promote the production of shikonin derivatives through metabolic engineering. Moreover, exploring the characteristics of AeHGO-catalyzed reaction will have profound implications for understanding the biosynthetic process of the naphthoquinone ring in shikonin derivatives.

## Nucleotide sequence accession numbers

The nucleotide sequences of AeHGO and unigene32185 have been deposited in the GenBank™ database under the accession numbers OP764689 and OP846989, respectively.

## Data availability statement

The datasets presented in this study can be found in online repositories. The names of the repository/repositories and accession number(s) can be found below: https://www.ncbi.nlm.nih.gov/genbank/, OP764689 https://www.ncbi.nlm.nih.gov/genbank/, OP846989.

## Author contributions

RW, SW, L. Guo, and LH conceived and designed research. RW, CZL, CGL, and JS conducted the most experiments. CK, YM, and JG analyzed the data. LS and JW maintained the plant materials. JG, and X. Wan helped discussing and designing experiments. RW wrote the manuscript. SW, JG, and L. Guo revised the manuscript. All authors contributed to the article and approved the submitted version.
