# Supplementary material for: The discovery and characterization of AeHGO in the branching route from shikonin biosynthesis to shikonofuran in Arnebia euchroma
Source: Front Plant Sci. 2023 Apr 25;14:1160571. doi: 10.3389/fpls.2023.1160571 (PMC10167036; doi:10.3389/fpls.2023.1160571)
Supplement: Supplementary file 1 [file DataSheet_1.docx]

Supplementary Material

**Figure S1.** Alignment of the encoded polypeptides of the candidate genes…………………….…..…..2

**Figure S2.** Functional characterization of the candidate genes….………………….…….………..…..3

**Figure S3.** Purification of AeHGO…...………………………………………………………....……..4

**Figure S4.** Purification of MgFR…………………………..………………………………….…....….4

**Figure S5.** ^1^H NMR spectrum of (*E*)-3''-oxo-GHQ in acetone-*d*_6_ (500 MHz)………………..….……..5

**Figure S6.** ^13^C NMR spectrum of (*E*)-3''-oxo-GHQ in acetone-*d*_6_ (125 MHz)………………..….…….5

**Figure S7.** ^1^H NMR spectrum of (*E*)-3''-OD-GHQ in acetone-*d*_6_ (500 MHz)………………..….……..6

**Figure S8.** ^1^H NMR spectrum of (*E*)-3''-OH-GHQ in acetone-*d*_6_ (500 MHz)………………..….……..6

**Figure S9.** Velocity versus substrate concentration plots of AeHGO-catalyzed reactions………..……7


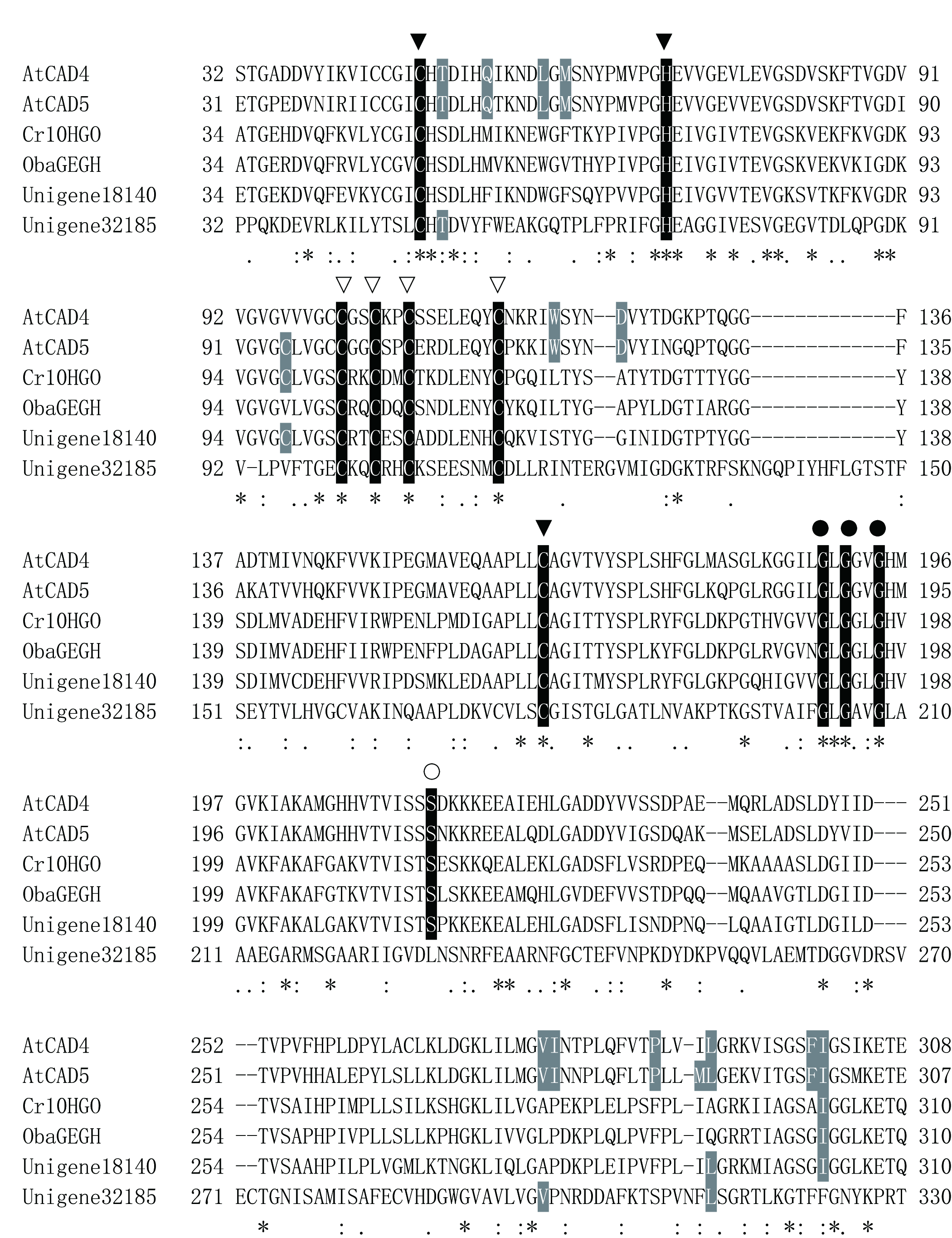


**Figure S1.** Alignment of the encoded polypeptides of the candidate genes. Amino acid sequences of reported CADs from *A. thaliana*, GEGH from *Ocimum basilicum* and 10HGO from *Catharanthus roseus* are used for comparison with candidate genes. Amino acids shared by the sequences are in black strips. The residues highlighted in grey are residues that were predicted to be important in AtCAD5 substrate binding. The binding sites of catalytic (solid triangle) and structural (hollow triangle) Zn ions are indicated, along with the G*X*G*XX*G sequence (black circle) and Ser (hollow circle) involved in determining cofactor specificity. The Genbank accessions for AtCAD4, AtCAD5, Cr10HGO, and ObaGEGH are NP_188576.1, NP_001031788.1, AHA82031.1 and Q2KNL6.1, respectively.


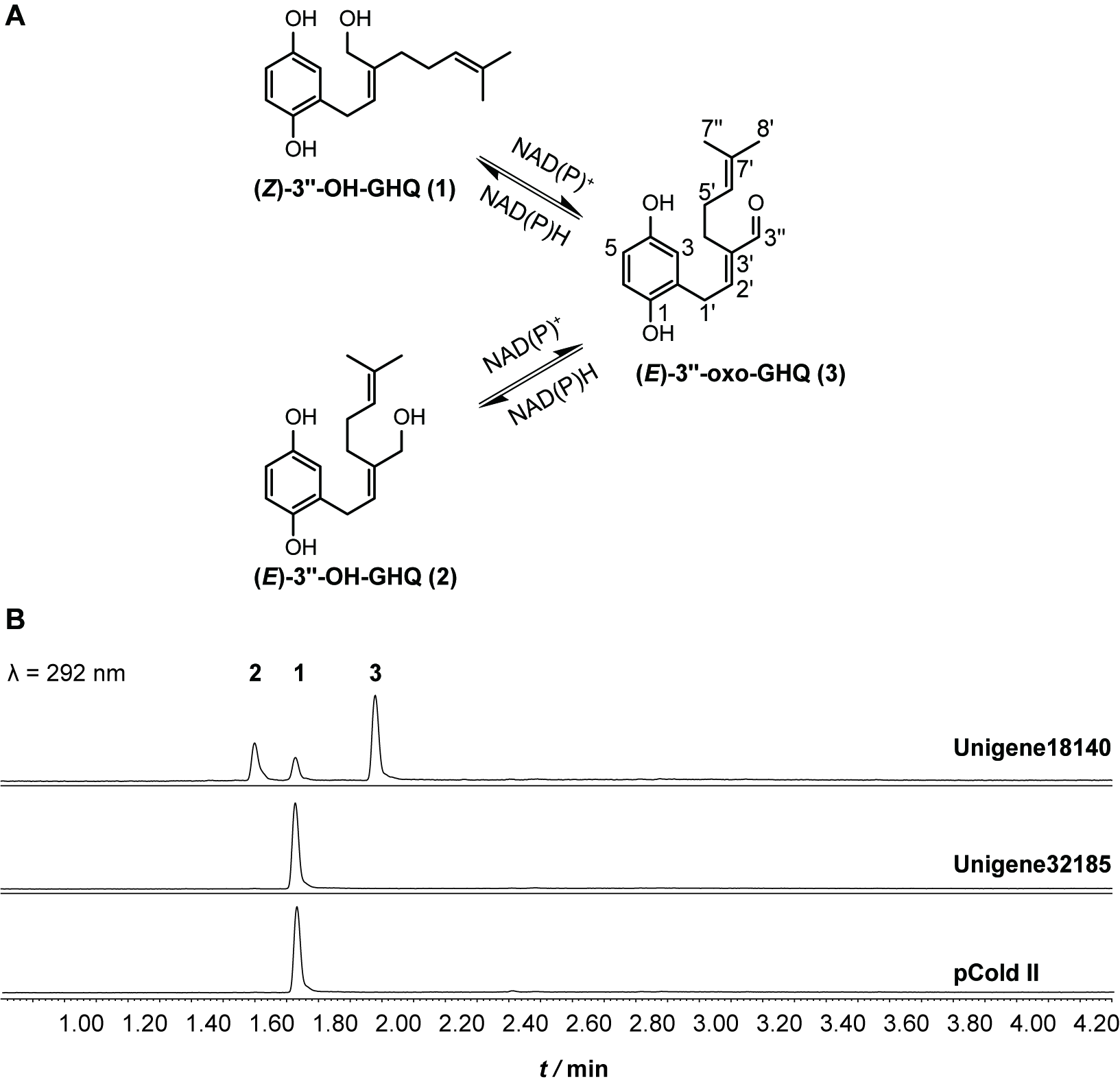


**Figure S2.** Functional characterization of the candidate genes. The chemical structures of the compounds and corresponding peaks in UPLC chromatogram are marked as **1**, **2**, and **3**. Crude enzyme from the *E. coli* carrying empty vector pCold II is used as a control. The detection wavelength was set at 292 nm. (A) Reaction catalyzed by the crude enzyme of unigene18140. (B) Incubation containing (*Z*)-3''-OH-GHQ (**1**) with the crude enzyme of unigene18140, unigene32185, and control respectively in the presence of NADP^+^.


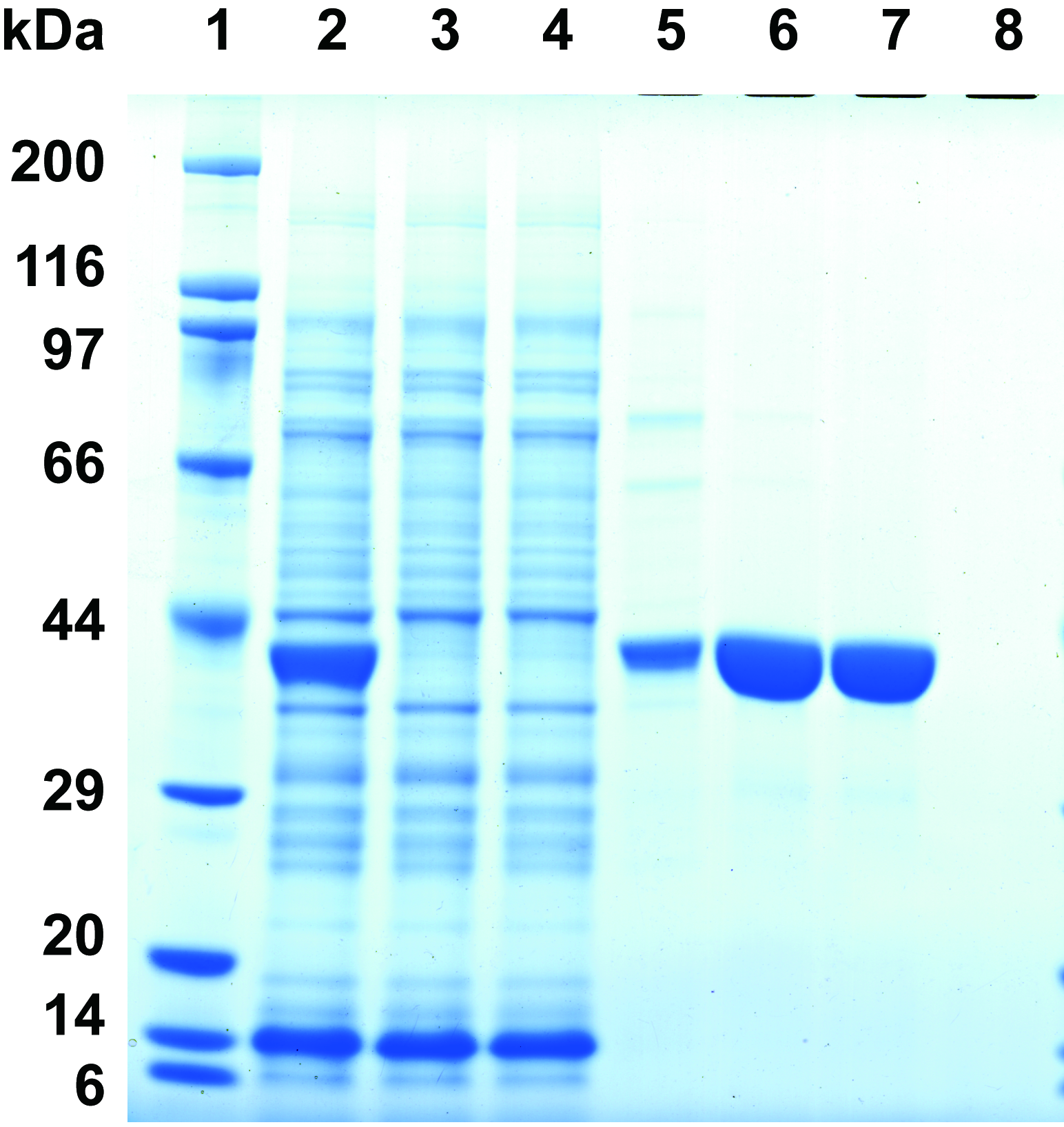


**Figure S3.** Purification of AeHGO. Lanes: 1, molecular mass ladder; 2, *Escherichia coli* crude extract; 3–8, fractions eluted from the metal chelate affinity column between 20 and 500 mM imidazole in NaH_2_PO_4_ buffer. Proteins were visualized by coomassie brilliant blue staining.


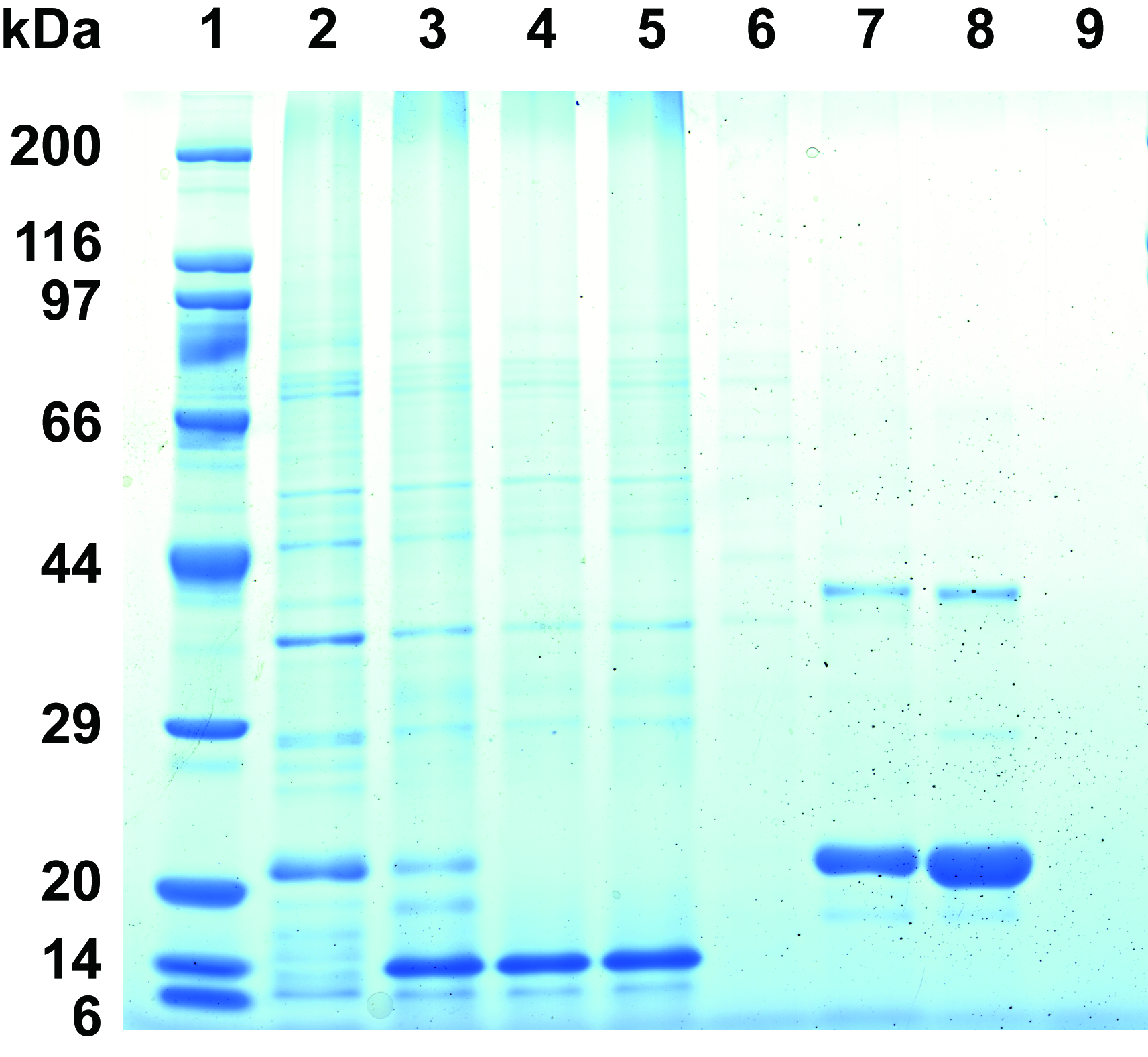


**Figure S4.** Purification of MgFR. Lanes: 1, molecular mass ladder; 2, *Escherichia coli* crude extract; 3–9, fractions eluted from the metal chelate affinity column between 20 and 500 mM imidazole in NaH_2_PO_4_ buffer. Proteins were visualized by coomassie brilliant blue staining.


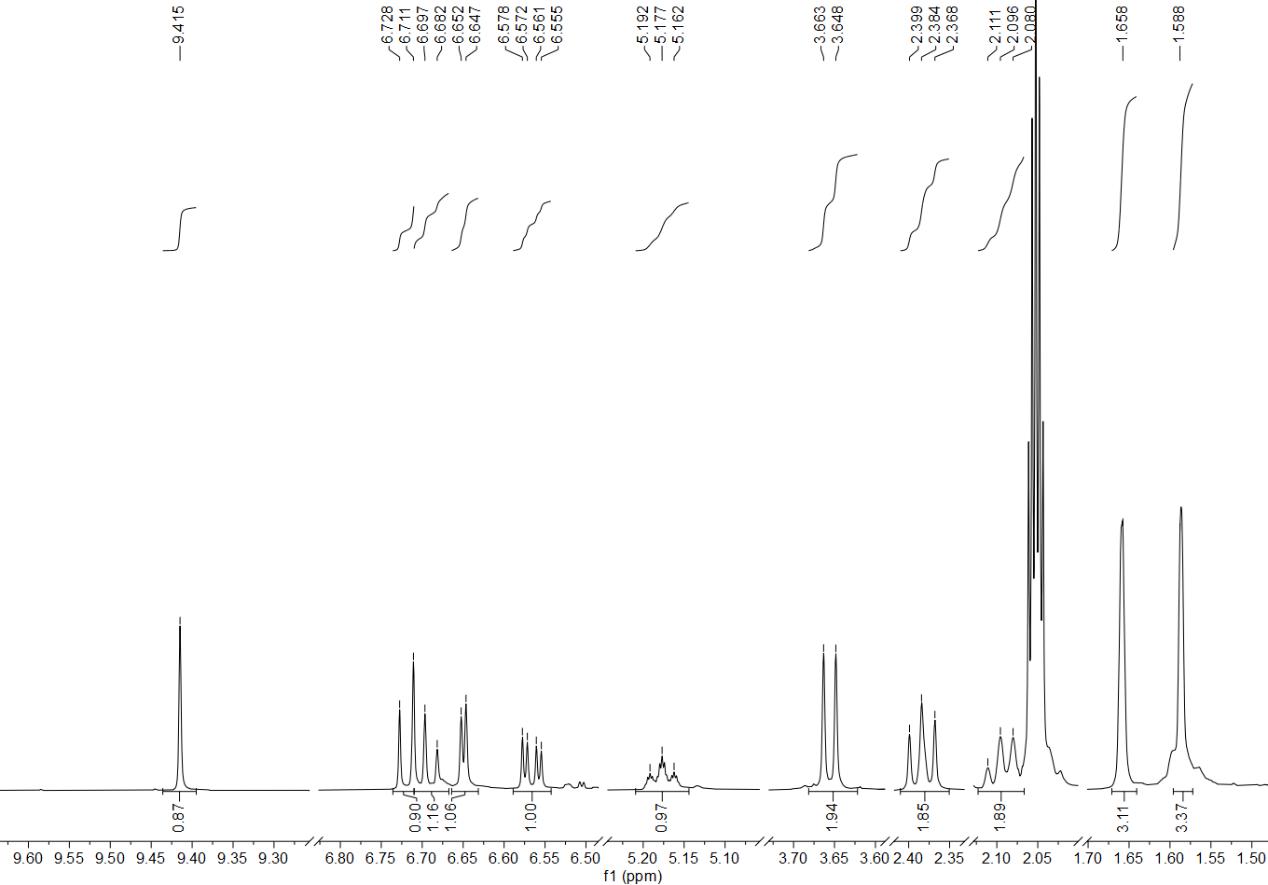


**Figure S5.** ^1^H NMR spectrum of (*E*)-3''-oxo-GHQ in acetone-*d*_6_ (500 MHz)


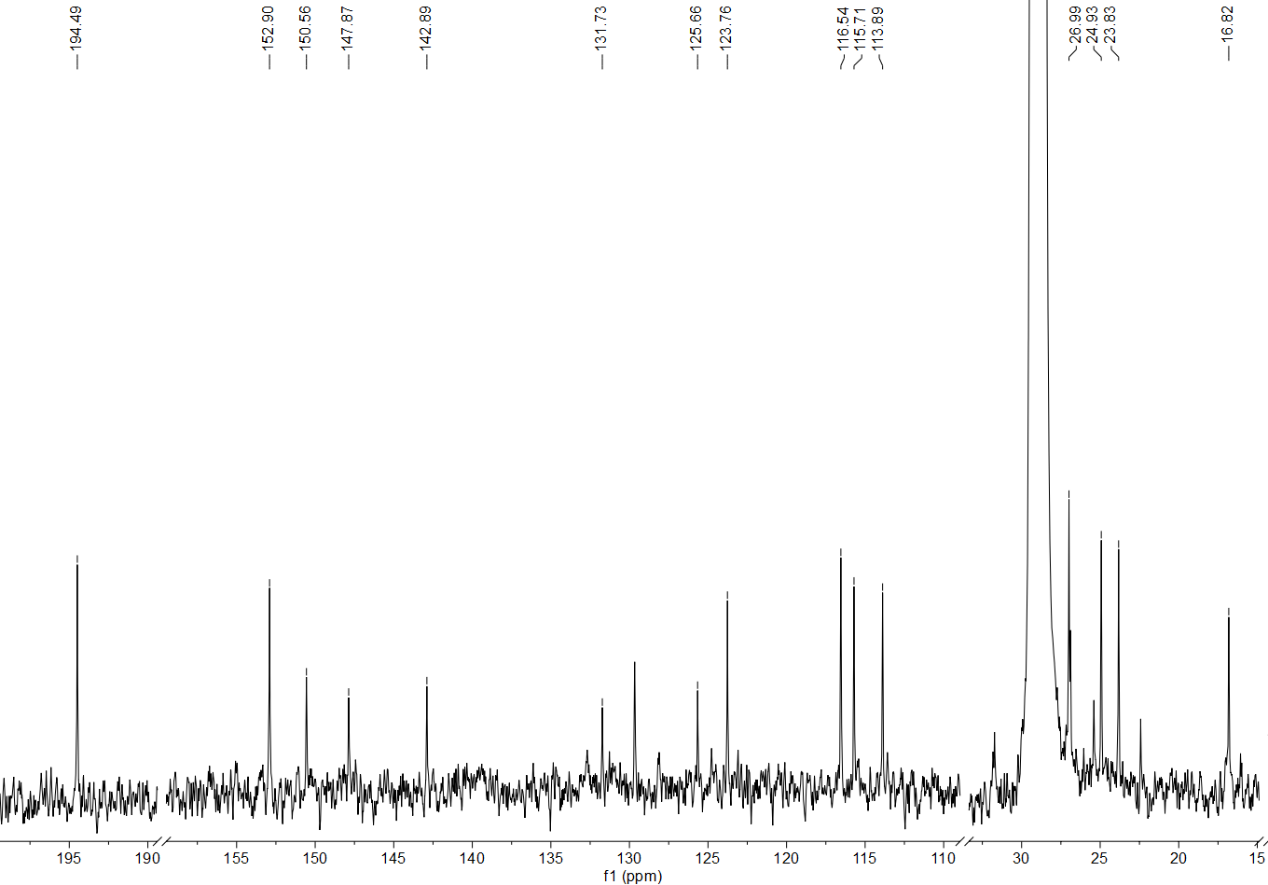


**Figure S6.** ^13^C NMR spectrum of (*E*)-3''-oxo-GHQ in acetone-*d*_6_ (125 MHz)


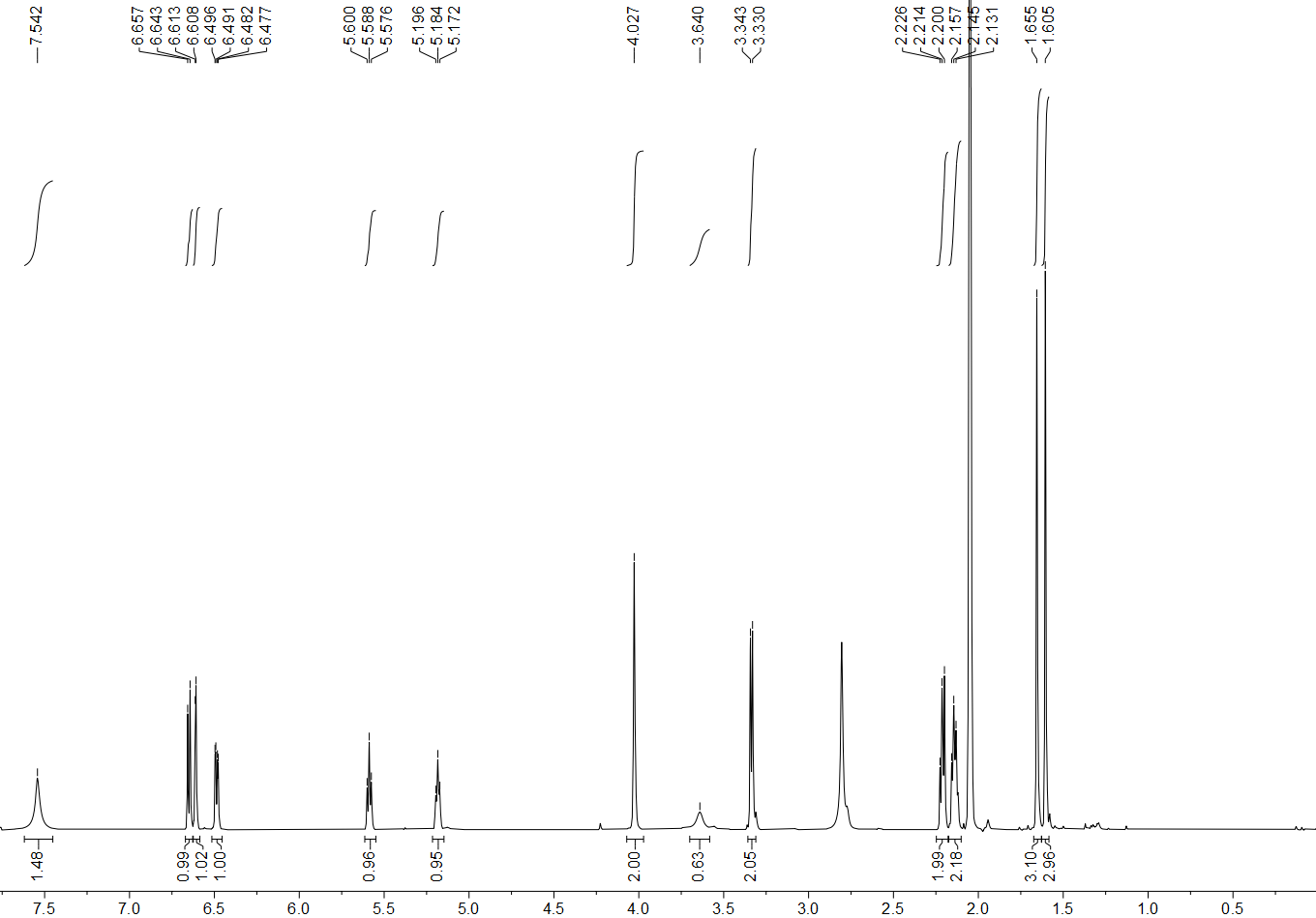


**Figure S7.** ^1^H NMR spectrum of (*E*)-3''-OD-GHQ in acetone-*d*_6_ (500 MHz)


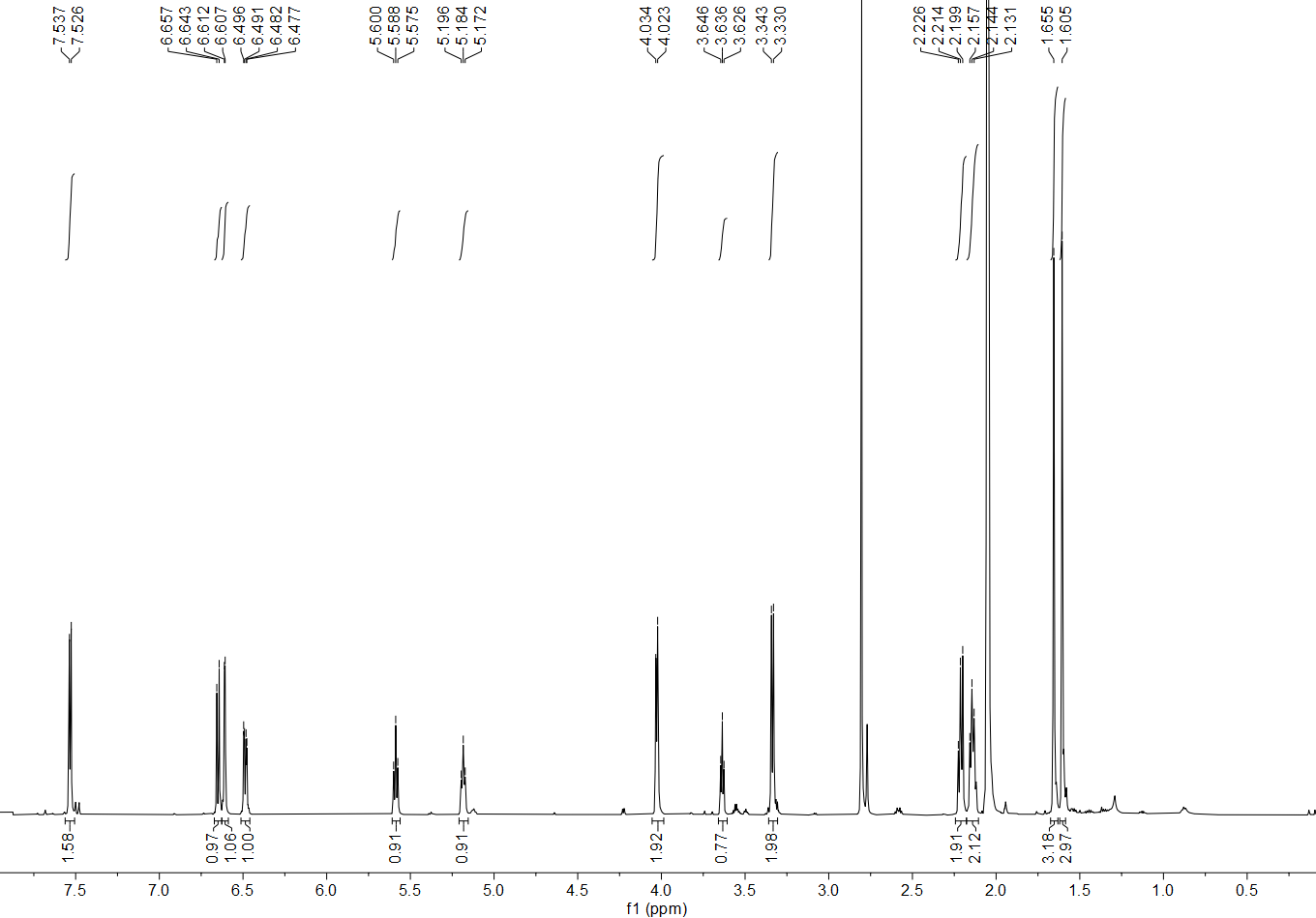


**Figure S8.** ^1^H NMR spectrum of (*E*)-3''-OH-GHQ in acetone-*d*_6_ (500 MHz)


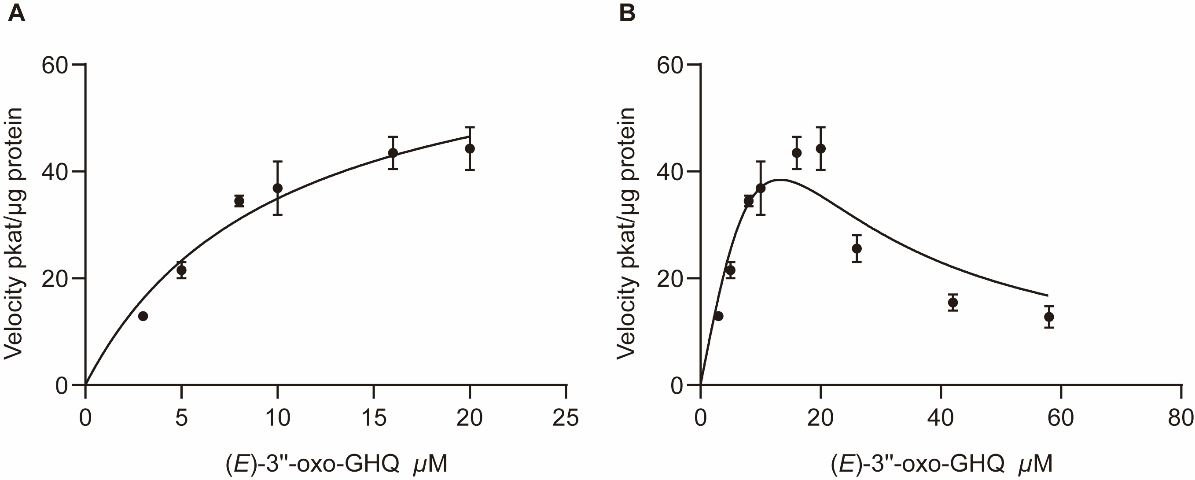


**Figure S9.** Velocity versus substrate concentration plots of AeHGO-catalyzed reactions. (A) AeHGO-catalyzed reduction of (*E*)-3''-oxo-GHQ (**3**) at low substrate concentrations. (B) The substrate inhibition occurring at concentrations exceeding 20 *µ*M.
